# *Borrelia burgdorferi* sensu lato Employs Several Escape Mechanisms to Bypass the Human Defense System

**DOI:** 10.1002/eji.70195

**Published:** 2026-05-04

**Authors:** Zara Karami, Hadewych J. M. ter Hofstede, Leo A. B. Joosten

**Affiliations:** ^1^ Department of Internal Medicine Radboud University Medical Center Nijmegen the Netherlands; ^2^ Radboud Community for Infectious Diseases (RCI) Radboud University Medical Center Nijmegen the Netherlands; ^3^ Department of Translational Immunology MedFuture Institute for Biomedical Research Iuliu Hatieganu University of Medicine and Pharmacy Cluj‐Napoca Romania

**Keywords:** Lyme borreliosis, *Borrelia burgdorferi*, Antigen presentation, Autophagy, Adaptive immune system

## Abstract

Lyme borreliosis (LB), caused by *Borrelia burgdorferi* sensu lato (*Bb*sl) through Ixodes tick bites, presents diverse clinical manifestations and may lead to persistent symptoms. This review summarizes current knowledge on the pathogen–host interactions and immune responses. Early infection can be influenced by tick saliva, which suppresses local host defense and promotes spirochete survival, and by pattern recognition receptors activating proinflammatory cascades. *Bb*sl employs a variety of immune evasion strategies, notably impairing antigen presentation—through disruption of MHC II and IFN‐γ pathways—and continuously varying surface antigens to hinder long‐lasting antibody formation. Autophagy plays a central role in modulating inflammation and T helper 17 adaptive immune responses, representing an underappreciated mechanism potentially influencing disease outcome. Adaptive immunity in LB is characterized by robust but often dysregulated humoral and cellular responses, with transient germinal centers and enduring IgM production contributing to incomplete pathogen clearance. Persistent immune defects include impaired long‐term B cell memory, suppressed T cell activation, and ongoing immunosuppression after pathogen clearance. Similar patterns are observed in other postinfectious fatigue syndromes. Despite advances, gaps remain in understanding mechanisms of *Bb*sl persistence and the immunopathology underlying chronic disease, challenging diagnosis and therapy. Emerging molecular and cellular approaches offer new avenues to address immunity, diagnostics, and prevention. A multidisciplinary effort will be needed to improve long‐term patient outcomes in the evolving epidemiology of LB.

## Graphical abstract

Integrated overview of host defense and immune evasion in Lyme Borreliosis. *Bb*sl evades immunity through tick saliva‐mediated suppression of innate defenses, TLR2/NOD2‐driven cytokine dysregulation, and interference with JAK/STAT and CIITA‐dependent antigen presentation. Impaired autophagy skews responses toward pathogenic Th17 activity, while disrupted lymph node architecture, persistent IgM, and defective CD4+ T cell function collectively impair long‐term IgG memory, facilitating chronic infection and immunopathology.

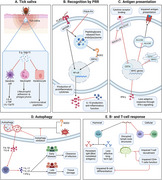

**Abbreviations**
APCs: antigen‐presenting cells
*Bb*sl: *Borrelia burgdorferi* sensu lato
CIITA: class II transactivator
CLR: C‐type lectin receptors
CRASP: complement regulator‐acquiring surface proteins
cQTL: chromatin quantitative trait locus
DC: dendritic cell
EM: erythema migrans
FH: factor H
FHL‐1: factor H‐like protein 1
FlaB, P41: Flagellin B, 41‐kDa
GCs: germinal centers
IFN‐γ: interferon gamma
IgG: immunoglobulin G
IgM: immunoglobulin M
IL: interleukin
LB: Lyme borreliosis
MAC: membrane attack complex
MHC: major histocompatibility complex
MYD88: myeloid differentiation primary response 88
NF‐κB: nuclear factor kappa‐light‐chain‐enhancer of activated B cells
NOD2: nucleotide‐binding oligomerization domain‐containing protein 2
OspA/OspC: outer surface protein A/C
PRRs: pattern recognition receptors
ROS: reactive oxygen species
SOCS1/SOCS3: suppressor of cytokine signaling 1/3
Th17: T helper 17 cell subset
TLRs: toll‐like receptors
TNF: tumor necrosis factor
VlsE: variable major protein‐like sequence expressed

## 1 Background

Lyme borreliosis (LB), the most common vector‐borne disease in the Northern Hemisphere, is caused by spirochetes of the *Borrelia burgdorferi* sensu lato (*Bb*sl) complex and is transmitted through the bite of infected *Ixodes* spp ticks [1]. Annually, it is estimated to account for approximately 476,000 cases in the United States and over 200,000 cases in Europe [2, 3, 4]. The clinical spectrum of LB is remarkably diverse. Early localized *Bb*sl infection is commonly identified by erythema migrans (EM), a bull's‐eye rash present in 70%–80% of LB cases [5]. From the initial skin site, the spirochete can disseminate systemically via hematogenous and lymphatic routes, potentially leading to early disseminated disease, late disseminated disease, or, in some cases, persistent disease [6].

While the initial infection is often characterized by localized inflammation, the progression of LB is largely dependent on the complex interplay between the pathogen and the host immune system [7, 8]. In this review, the different host defense mechanisms: innate immune recognition, antigen presentation, autophagy, and adaptive immune response, will be highlighted. Pattern recognition receptors (PRRs), including Toll‐like receptors (TLRs) and NOD‐like receptors, play a pivotal role in detecting *Bb*sl components and initiating the inflammatory cascade [9, 10, 11, 12]. Subsequent antigen presentation by dendritic cells and macrophages bridges innate and adaptive immunity, shaping the quality and magnitude of T and B cell responses [13, 14]. Autophagy, a cellular process for degrading and recycling intracellular components, has emerged as a key regulator of both pathogen clearance and immune modulation in LB [15, 16]. The adaptive immune response—encompassing both cellular (T cell‐mediated) and humoral (antibody‐mediated) mechanisms—can determine the outcome of infection, persistence of the pathogen, and the risk of chronic inflammation or autoimmunity [17, 18, 19, 20].

Despite advances in understanding these immunological processes, significant gaps remain in our knowledge of how *Bb*sl manipulates host immunity to evade clearance and promote dissemination, and in some patients, persisting symptoms after treatment. This review summarizes the current insights into PRR signaling, antigen presentation, autophagy, and adaptive immune responses in LB, highlighting emerging concepts and unresolved questions. By discussing these mechanisms, we aim to gain more insights for future research directions and support the development of more effective diagnostic and therapeutic strategies for LB.

## 2 The Infection Process and Early Host Response

After the bite of an infected Ixodes tick, *Bb*sl is introduced into the human skin together with immunomodulatory substances present in the tick's saliva. These molecules suppress early local immune responses, specifically inhibiting the recruitment and function of neutrophils at the site of infection, which allows the spirochetes to survive and proliferate in the dermis [21, 22, 23]. The first host cells in the skin that encounter *Bb*sl are keratinocytes, fibroblasts, tissue‐resident macrophages, dendritic cells, and infiltrating neutrophils [24, 25]. Keratinocytes themselves can produce antimicrobial peptides and inflammatory mediators, but their responses are inhibited by tick‐derived proteins, such as Salp15, further delaying effective pathogen clearance [23, 26, 27]. Another effect of Salp15 is the inhibition of dendritic cell secretion of IL‐6, TNF, and IL‐12p70 [28]. The spirochetes also co‐opt local extracellular matrix components such as decorin‐binding adhesins, fibronectin, and integrins to adhere and persist within the skin and to facilitate migration through tissues [29, 30, 31, 32].

Initial recognition of *Bb*sl is mediated by pattern recognition receptors (PRRs) such as Toll‐like receptor 2 (TLR2), NOD‐like receptor 2 (NOD2), and C‐type lectin receptors (CLR) on innate immune cells like macrophages, dendritic cells, and neutrophils [33]. Among these, TLR2, often in combination with either TLR1 or TLR6, is central for detecting the abundant lipid‐modified outer membrane proteins unique to the spirochete [9, 10, 11]. This TLR2‐dependent signaling initiates MYD88‐dependent signaling, activating NF‐κB and caspase‐1, which together drive transcription and release of proinflammatory cytokines such as IL‐1β, TNF, and IL‐6 [34, 35]. NOD2 is responsible for detecting peptidoglycans and activating NF‐κB and cytokine transcription of IL‐1β, IL‐6, IL‐10, and TNF [12]. NOD2 promotes inflammation in the early phase of infection but subsequently acts to restrain chronic tissue inflammation by inducing tolerance [36]. In Table 1, different innate immune cells are highlighted with their primary PRR and major cytokines produced. Phagocytosis of live spirochetes by macrophages further amplifies transcription of proinflammatory genes and activates inflammasome pathways [37]. Interestingly, intact bacteria trigger stronger immune activation than *Bb*sl lysates, indicating that specific internalization and trafficking events drive unique signaling cascades [10].

**TABLE 1:** Overview of principal innate immune cells involved in early host responses to *Bb*sl. With every innate immune cell, the corresponding pattern recognition receptors (PRRs), the main cytokines produced, and key *Bb*sl immune evasion strategies are mentioned.

Innate immune cell	Key PRRs	Major induced cytokines	*Bb*sl evasion strategy
Keratinocyte	TLR2, TLR1, NOD2	IL‐8, IL‐6, TNF, CXCL1	Inhibition by tick saliva (Salp15) blocks cytokine and antimicrobial peptide production; suppresses early inflammatory signaling
Fibroblast	TLR2, NOD2	IL‐6, IL‐8, CXCL1	Tick salivary gland extract (SGE) can have cytotoxic actions on fibroblasts at higher concentrations, causing cell death Tick saliva may also hinder fibroblast migration and wound repair by inhibiting key growth factors (PDGF, EGF, TGF‐α)
Macrophage	TLR2, TLR1, NOD2	TNF, IL‐1β, IL‐6, IL‐10	Recruitment of complement factor H/fH‐like to block opsonization; induction of IL‐10 to suppress inflammatory responses; resisting phagocytosis
Dendritic cell	TLR2, TLR1, NOD2	IL‐12, IL‐6, TNF, IL‐10	Impaired maturation by Salp15 and IL‐10; inhibition of MHC II expression and antigen processing; altered cytokine milieu
Neutrophil	TLR2, TLR1, NOD2	IL‐8, TNF, IL‐1β, ROS	Tick saliva inhibits neutrophil recruitment/function; *Bb*sl evades killing via complement resistance and modulates chemotaxis

**FIGURE 1:**
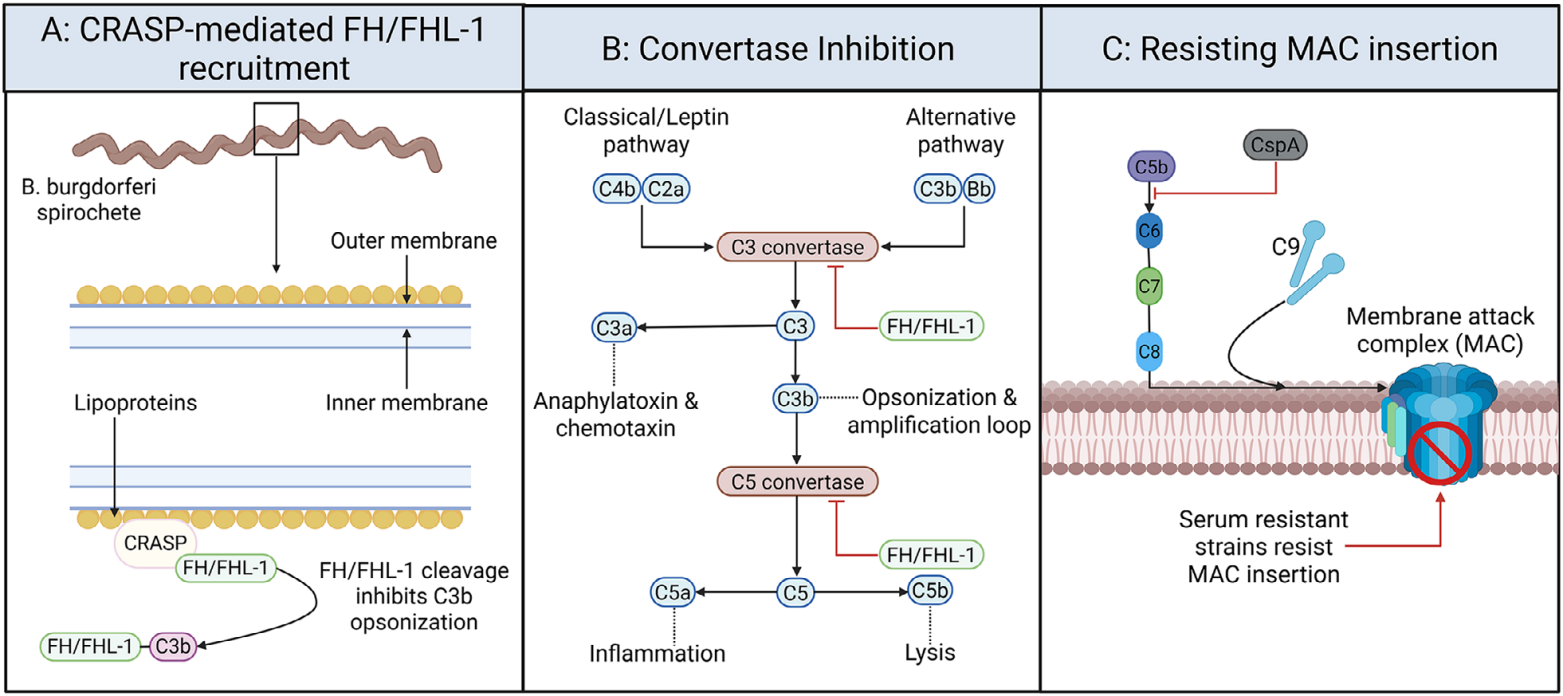
Complement evasion mechanisms by *Bb*sl. (A) *Bb*sl surface‐expressed CRASPs bind circulating regulators FH and FHL‐1, which act as cofactors for factor I‐mediated C3b cleavage, thereby blocking opsonization. (B) FH/FHL‐1 binding prevents assembly of C3 and C5 convertases via both the classical (C4bC2a) and alternative (C3bBb) pathways, inhibiting the further development of the complement system. (C) Serum‐resistant strains prevent insertion of the C5b‐9 MAC into their outer membrane through surface modifications.

Coinciding with these proinflammatory mechanisms, *Bb*sl evades immune elimination through a repertoire of strategies, of which complement resistance is particularly well characterized [38]. Complement evasion is a central mechanism enabling *Bb*sl to survive in the host bloodstream and evade early innate killing (Figure 1). At the molecular level, *Bb*sl expresses a family of complement regulator‐acquiring surface proteins (CRASP‐1 to CRASP‐5), which bind and recruit host fluid‐phase complement regulators, including factor H (FH) and FH‐like protein 1 (FHL‐1), to the bacterial surface [39, 40, 41]. FH and FHL‐1 function as cofactors for factor I‐mediated cleavage of C3b, thereby inhibiting C3 and C5 convertase formation and preventing downstream complement activation. In addition, *Bb*sl can resist insertion of the membrane attack complex (MAC) through surface modifications, further enabling survival in serum [38, 42, 43]. These mechanisms are strain‐specific. *Bb*sl isolates differ in their susceptibility to complement‐mediated killing. Serum‐resistant species, including *B. afzelii* and certain *B. burgdorferi* sensu stricto strains, express distinct CRASP profiles compared with serum‐sensitive strains. This underscores the importance of complement evasion for survival in the host [44, 45, 46]. Beyond complement, *Bb*sl induces production of IL‐10, an anti‐inflammatory cytokine that counterbalances strong innate activation [47, 48, 49]. Such immune modulation promotes persistence during critical days following tick detachment [50, 51, 52, 53].

While most spirochetes initially remain clustered in the dermis, *Bb*sl can disseminate through lymphatic vessels and the bloodstream [54]. The process of early spread is shaped by the cytokine and chemokine networks established in the skin, which can either restrict bacterial trafficking or, conversely, facilitate dissemination by recruiting permissive immune subsets [55]. Migration through the extracellular matrix and entry into lymphatics occur within days, allowing spirochetes to access distant tissues, including the heart, joints, and nervous system [6, 56]. Altogether, the infection process and early host response to *Bb*sl can be understood as a dynamic race. The balance of these early interactions strongly influences whether the infection remains localized or progresses to disseminated disease.

## 3 Antigen Presentation and Adaptive Immunity

Following the initial innate immune responses at the site of *Bb*sl infection, antigen presentation emerges as a pivotal step in bridging innate detection and activation of adaptive immunity [13, 57]. Antigen‐presenting cells (APCs), predominantly dendritic cells and macrophages, internalize and process *Bb*sl antigens to present them on major histocompatibility complex (MHC) molecules [58]. These cells migrate to draining lymph nodes where they prime naïve T cells, a prerequisite for effective B cell help, germinal center (GC) formation, and cytotoxic effector responses [57]. Understanding how *Bb*sl subverts this process is therefore essential, because defects introduced at the level of the APC propagate causally through every downstream arm of adaptive immunity.

Mechanistically, *Bb*sl impacts antigen presentation through several complementary strategies across both professional APCs and local tissue cells. The spirochete inhibits the transcriptional activator CIITA in dendritic cells, leading to impaired expression of MHC II molecules necessary for effective CD4+ T cell activation. It also upregulates suppressors of cytokine signaling genes (SOCS1, SOCS3), attenuating IFN‐γ signaling—a critical regulator of antigen processing and MHC II upregulation in dendritic cells, macrophages, and even antigen‐presenting fibroblasts [59]. It should be noted that much of the evidence for CIITA inhibition is based on mRNA expression data. Some studies have reported increased rather than decreased MHC II surface expression in response to *Bb*sl antigens, and further functional studies are needed to reconcile these observations. *Bb*sl modulates antigen processing within APCs, with evidence for altered phagosomal signaling and downstream effects on antigen loading pathways [10, 14]. However, the precise impact on endosomal and lysosomal MHC II peptide loading remains to be fully characterized.

Recent advances have identified nontraditional antigen‐presenting cellular subsets, including antigen‐presenting fibroblasts within affected tissues such as skin and synovium. These fibroblasts can upregulate MHC II and co‐stimulatory molecules in response to IFN‐γ, potentially participating in local T cell activation and immune modulation within inflamed tissue sites [60, 61, 62, 63]. This emerging understanding underscores the complexity of antigen presentation beyond classic professional APCs in LB.

The direct consequence of this APC dysfunction is weak and quantitatively defective T cell priming. During the acute phase, lymphocytes from patients do show proliferative responses to key *Bb*sl antigens, including FlaB (P41 flagellin), OspC, and OspA. The frequency and diversity of these antigen‐specific responses increase during convalescence, with robust T cell reactivity against OspC and OspA [18]. However, these responses are generated in the context of an APC environment in which MHC II expression is suppressed, IFN‐γ signaling is attenuated, and co‐stimulatory molecule expression is diminished—conditions that inherently limit the quality of T cell activation. Compounding this, *Bb*sl induces APCs to secrete IL‐10. Studies in IL‐10 knockout mice demonstrate that endogenous IL‐10 restrains joint inflammation during *Bb*sl infection in vivo [47, 64]. Experiments in macrophage cell lines treated with exogenous IL‐10 confirm that this cytokine suppresses proinflammatory mediators, including TNF, IL‐6, and IL‐12, reduces co‐stimulatory molecule expression (with the exception of CD86), and diminishes phagocytic capacity [65]. However, the extent to which exogenous IL‐10 in vitro faithfully recapitulates endogenously produced IL‐10 during natural infection should be interpreted with caution. Collectively, this immunosuppressive APC environment restricts the T cell activation required for productive downstream lymphoid responses.

Impaired T cell priming likely contributes to defective GC formation and disrupted lymph node architecture. *Bb*sl infection causes a loss of organized T and B cell zones within lymph nodes [66]—a phenomenon that, while particularly prominent in LB, is also observed in many acute viral and bacterial infections and may thus reflect a general feature of robust inflammatory responses rather than *Bb*sl‐specific effect. CD4^+^ T cells, including T follicular helper cells, are induced during *Bb*sl infection but support only transient GC responses and short‐lived antibody production, indicating qualitatively altered follicular help rather than sustained GC support [67, 68]. Moreover, single‐cell transcriptomic analyses reveal early extrafollicular B cell proliferation with isotype switching in the absence of fully developed germinal centers, further reflecting an atypical antibody response development [55].

With GC reactions compromised, the humoral response to *Bb*sl is marked by persistent IgM production that far outlasts what is seen in self‐limiting bacterial infections such as those caused by *Streptococcus pneumoniae* or *Staphylococcus aureus—*where IgM production typically wanes within weeks—and is accompanied by comparatively less effective generation of long‐term IgG‐mediated immunity [69, 70]. The antibodies produced—both T cell‐independent and T cell‐dependent—target multiple surface proteins but exhibit only transient affinity maturation, with persistent IgM and loss of high‐affinity IgG consistent with poorly sustained GC reactions [69, 71]. These qualitative deficiencies mean that, although antibodies can reduce bacterial load and provide some protective effect, they typically fail to eliminate the pathogen altogether [69, 70]. *Bb*sl aggravates this humoral insufficiency by continuously varying VlsE through segmental gene conversion at the vls locus and by downregulating OspC as VlsE is upregulated during mammalian infection [72, 73]. Furthermore, *Bb*sl infection suppresses antibody responses even to unrelated antigens, such as SARS‐CoV‐2 spike or influenza vaccine antigens [70, 74]. This suppression can persist after antibiotic treatment, with sustained elevations of antigen‐specific IgM despite restoration of near‐baseline IgG response [69, 74].

The nonredundant importance of humoral and cellular arms is hinted at by observations in immunocompromised patients. Individuals with impaired antibody production or combined immunodeficiencies appear more prone to disseminated or treatment‐refractory LB, although data are limited and largely derive from case reports [75, 76, 77]. These clinical observations support the use of immunocompromised patient cohorts as natural experiments to dissect the immune requirements for infection control.

**FIGURE 2:**
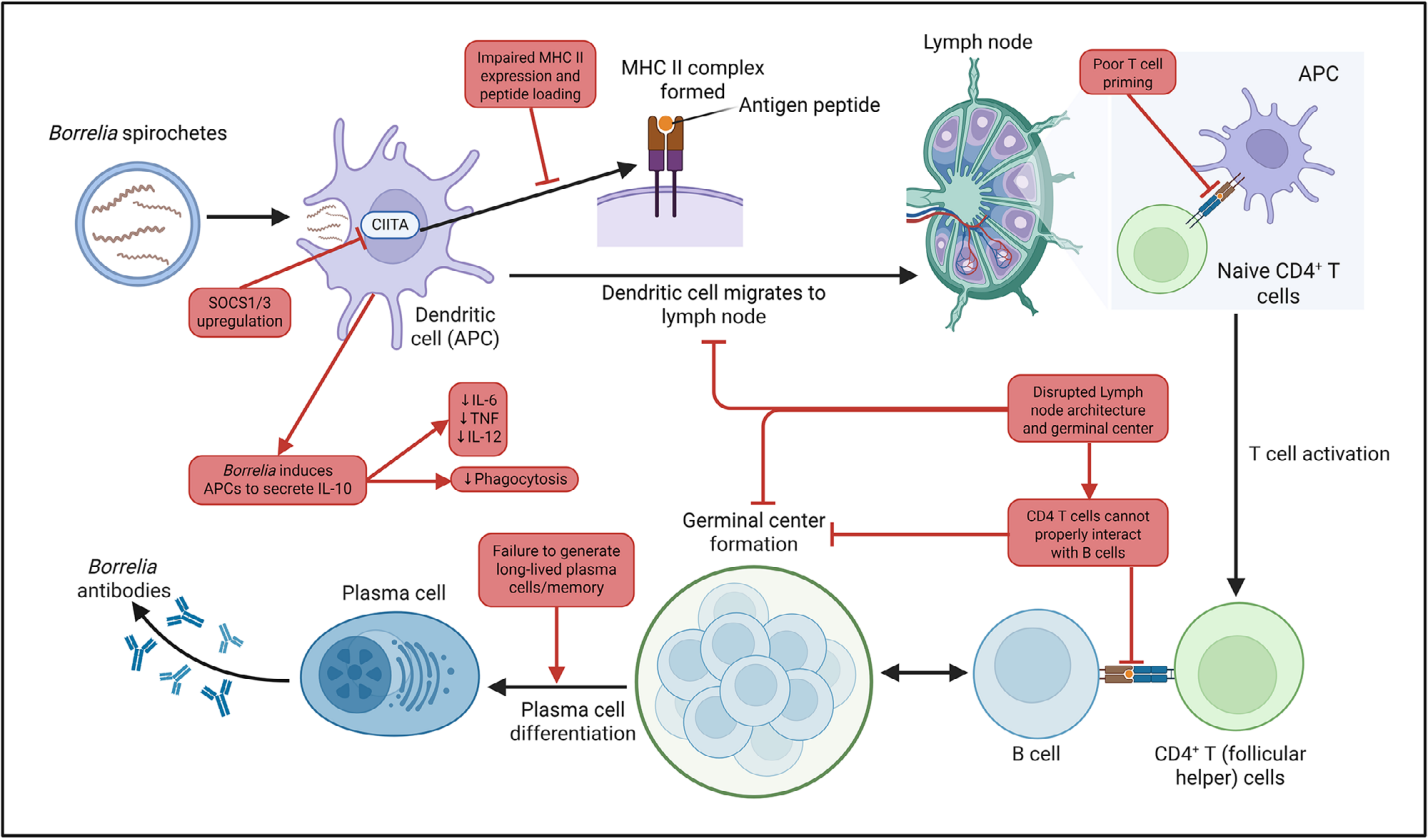
*Bb*sl interference with antigen presentation and adaptive immunity. Upon encountering *Bb*sl spirochetes, DCs process antigens via CIITA‐driven MHC class II expression for presentation to naïve CD4+ T cells. *Bb*sl subverts this at multiple levels: upregulation of SOCS1/3 impairs CIITA activity, reducing MHC II expression and antigen peptide loading; IL‐10 induction suppresses pro‐inflammatory cytokine production and phagocytosis; and disruption of lymph node and germinal center architecture impairs DC–T cell interactions, resulting in poor CD4^+^ T cell priming. Consequently, CD4^+^ T cell–B cell interactions are compromised, germinal center formation is disrupted, and plasma cell differentiation fails to generate long‐lived memory. Black arrows indicate the normal immune response; red arrows and blunt‐ended lines indicate *Bb*sl‐mediated inhibition or dysregulation. APC, antigen‐presenting cell; CIITA, class II major histocompatibility complex transactivator; MHC, major histocompatibility complex; SOCS, suppressor of cytokine signaling.

Overall, data suggest that antigen presentation in LB does not fail outright but is skewed in ways that promote dysregulated, largely nonsterilizing adaptive responses [68, 78]. A schematic overview of how *Bb*sl interferes with antigen presentation and the adaptive immune response is shown in Figure 2. At the same time, both patients and experimental models clearly mount strong T‐cell and multiantigen antibody responses, indicating that the defect lies in the quality and organization of adaptive immunity rather than its absence [18, 68]. A critical unresolved issue is how specific DC subsets contribute to this balance: antigen‐pulsed DCs and skin DCs can prime protective immunity in experimental models, yet the in vivo roles and lymph‐node trafficking of defined DC subsets in LB remain comparatively underexplored, particularly with modern genetic fate‐mapping and depletion tools [79]. Resolving this gap will help pinpoint where along the APC‐T cell–B cell axis *Bb*sl most consequential immune evasion occurs, and thus which cellular nodes are most promising for targeted intervention.

## 4 Autophagy

**FIGURE 3:**
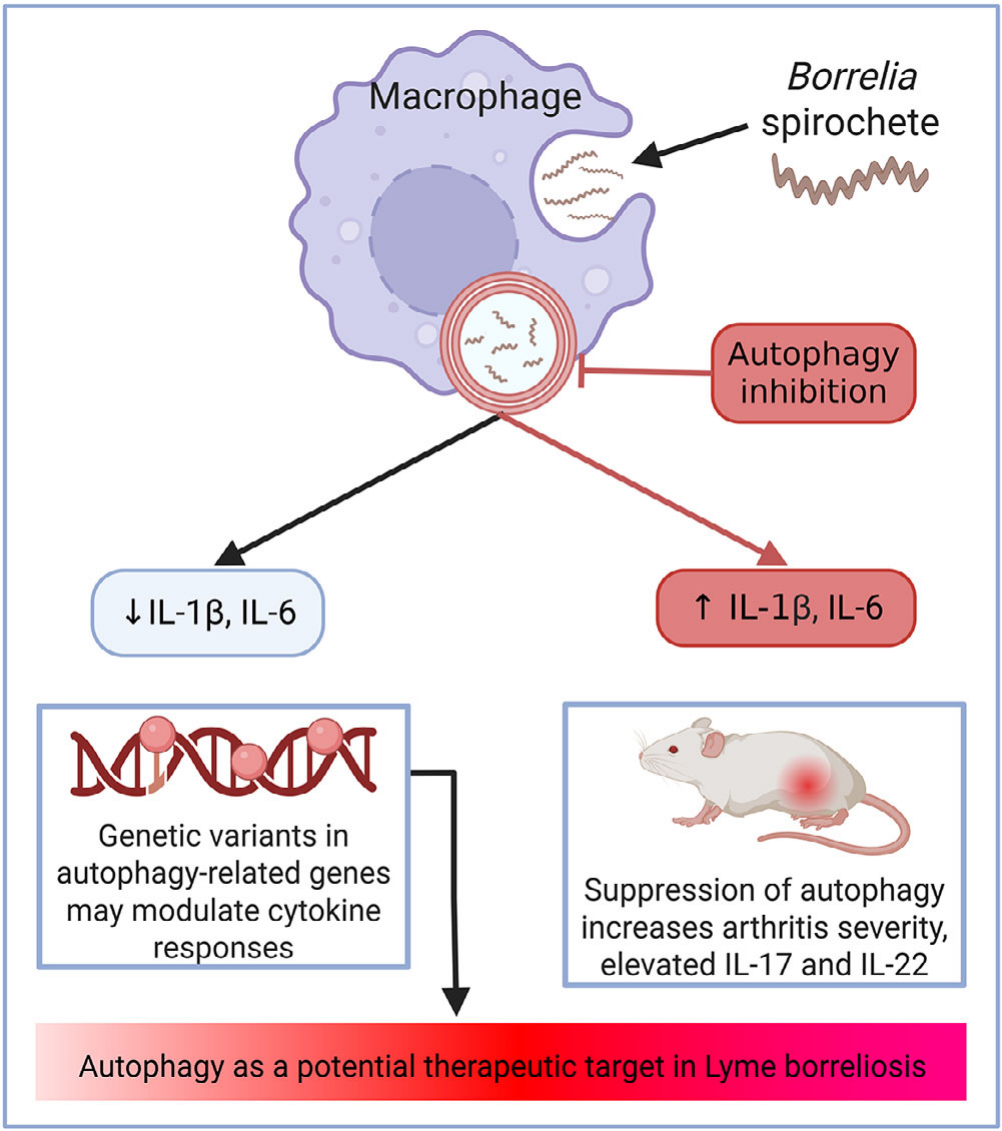
Autophagy regulation in Lyme borreliosis. Upon internalization of *Bb*sl spirochetes, macrophages sequester the bacteria within autophagosomes, reducing pro‐inflammatory cytokine production. Pharmacological inhibition of autophagy reverses this, driving elevated IL‐1β and IL‐6, and increasing arthritis severity with elevated IL‐17 and IL‐22 in murine models. Genetic variants in autophagy‐related genes may additionally modulate cytokine responses in LB patients. Together, these findings identify autophagy as a potential therapeutic target. Black arrows indicate active autophagy; red arrows indicate autophagy inhibition.

Studies have illuminated a pivotal role for autophagy in the immunological landscape of LB. Autophagy, an intracellular degradation process, is initiated in host immune cells upon exposure to *Bb*sl and serves as a key regulatory mechanism in controlling inflammation (Figure 3). Specifically, the induction of autophagy reduces the production of IL‐1β. Inhibition of autophagy—either pharmacologically or genetically—leads to heightened IL‐1β and IL‐6 release in response to *Bb*sl, while TNF concentrations remain unaffected. These effects are mediated at the transcriptional level and are dependent on reactive oxygen species, as patients with defects in ROS generation demonstrate exaggerated IL‐1β responses when autophagy is impaired [15, 16].

Experimental murine models further reveal that suppression of autophagy increases the severity of Lyme arthritis, characterized by greater joint swelling, cellular infiltration, and elevated cytokine production. Notably, the breakdown of autophagic regulation enhances secretion of IL‐17, IL‐22, and IFN‐γ, linking disordered autophagy to pathogenic Th17 immune responses. The increase in IL‐17 is specifically attributed to enhanced IL‐1β and is independent of IL‐23, marking a unique cytokine axis relevant to LB pathology [15].

Recent studies further reveal that genetic variants in autophagy‐related genes can shape *Bb*sl‐induced cytokine responses in LB patients. Upregulation and downregulation of core autophagy genes in response to infection support a link between autophagy regulation and individual variability in inflammatory outcomes. These discoveries position autophagy as a potential pathway influencing both disease severity and immune heterogeneity in LB [80] [under review]. More broadly, xenophagy has been shown to mediate intracellular killing of bacteria such as *Mycobacterium tuberculosis and Salmonella* [81, 82]. By analogy, autophagy‐linked pathways might also contribute to controlling *Bb*sl during this transient intracellular residence in phagocytosis, although this remains to be tested directly. Beyond rare variants in core autophagy genes, common cytokine pathway variants also strongly shape the host immune response to *Bb*sl. A recent cQTL study mapped numerous loci controlling ex vivo *Bb*sl‐induced cytokine production, linked several to LB susceptibility. These findings indicate that interindividual inflammatory set‐points are partly genetically hard‐wired and support genetically informed risk stratification and host‐directed therapies in LB [83].

Collectively, these findings suggest that autophagy acts as a brake on excessive inflammation and immune activation during LB. Its regulatory influence on cytokine networks, particularly the IL‐1β–Th17 axis, points to autophagy as a potential therapeutic target for mitigating tissue damage and chronic symptoms in LB.

## 5 Current knowledge gaps and future directions

Despite decades of research, many critical questions about LB remain unanswered, and the accelerating challenges of persistent and chronic disease highlight the urgency of innovative solutions. Promising new directions center around unraveling the mechanisms underlying *Bb*sl persistence, with advanced molecular and imaging tools now making it possible to visualize tissue and cellular niches where spirochetes evade immune clearance [84]. High‐throughput single‐cell and multiplex profiling are set to reveal which cell types and microenvironments support ongoing infection and immunological dysfunction, enabling more targeted therapeutic strategies [55].

A renewed focus on restorative immunology holds great potential [7]. Repairing damaged germinal center architecture and reversing lymphoid tissue disorganization—perhaps by manipulating local cytokine milieus and chemokine pathways—could pave the way for more durable humoral memory and better resolution of infection [85]. Expansion in the field of autophagy also offers opportunities to dampen excessive inflammation and mitigate chronic symptoms, as new pharmacological and gene‐editing approaches emerge [86].

Continued progress against antigenic variation remains vital for vaccine and diagnostic development. Cutting‐edge protein engineering, epitope mapping, and AI‐driven immune profiling are merging to outpace *Bb*sl's molecular shape shifting, promising broader and more reliable detection, and improved vaccine design that can account for diverse strains and immune responses [87, 88, 89].

Integrated “‐omics” studies are beginning to yield predictive biomarkers for chronicity, offering hope that early risk stratification may soon be possible [90, 91]. This, alongside the rollout of multiplex rapid diagnostic tests and flexible laboratory platforms, is expected to transform both point‐of‐care management and long‐term disease tracking in endemic areas [92, 93].

Genetic studies continue to elucidate susceptibility to disease and long‐term complications, opening avenues for precision medicine and individualized prevention [94]. Functional genomics defining the QTL architecture of *Bb*sl‐induced cytokine responses illustrates the impact of common genetic variants in immune and metabolic pathways. These variants program individual inflammatory set‐points in LB [95]. As climate change and ecosystem alterations extend tick habitats, coordinated surveillance and prevention strategies will be crucial to reduce new infections and recurrences [96, 97].

Altogether, the future of LB research lies in bridging gaps across science, translational modeling, and patient‐centered clinical interventions. Only through a multidisciplinary and global approach can the field respond to the evolving challenges of diagnosis, treatment, and prevention, ultimately restoring effective immunity and improving outcomes for individuals affected by this complex disease.

## Author Contributions

Zara Karami: conceptualization, literature search, writing of the original draft, and preparation of figures. Hadewych ter Hofstede: supervision, critical revision of the manuscript, and final approval of the submitted version. Leo Joosten: conceptualization, supervision, critical revision of the manuscript, and final approval of the submitted version.

## Conflicts of Interest

The authors declare no conflicts of interest.

## Data Availability

The manuscript does not contain original animal experiments or human studies. Data are available upon request.
